# Cucurbitacin D Overcomes Gefitinib Resistance by Blocking EGF Binding to EGFR and Inducing Cell Death in NSCLCs

**DOI:** 10.3389/fonc.2020.00062

**Published:** 2020-02-18

**Authors:** Se Hyang Hong, Jin Mo Ku, Ye Seul Lim, Seo Yeon Lee, Ji Hye Kim, Chunhoo Cheon, Seong-Gyu Ko

**Affiliations:** ^1^Department of Preventive Medicine, College of Korean Medicine, Kyung Hee University, Seoul, South Korea; ^2^Department of Science in Korean Medicine, Graduate School, Kyung Hee University, Seoul, South Korea

**Keywords:** EGFR, NSCLC, drug resistance, lung adenocarcinoma cancer, cucurbitacin D

## Abstract

In this study, the mechanism of the anticancer effect through which cucurbitacin D (CuD) can overcome gefitinib resistance in NSCLC was investigated. Cell viability was measured by 3-(4,5-dimethylthiazol-2-yl)-2-5-diphenyltetrazolium bromide assay, and cell migration and growth were observed by wound healing and colony formation assays, respectively. Levels of EGFR family members, protein kinase B, extracellular signal-regulated kinase, poly(ADP-ribose) polymerase, and G2/M phase-related proteins were detected by Western blot analysis. Immunofluorescence analysis was used to detect the intracellular expression of p-EGFR. Induction of apoptosis and cell cycle arrest was measured by flow cytometry. Solid-phase binding assays were used to determine binding to the EGFR family. CuD inhibits the phosphorylation of EGFR in gefitinib-resistant NSCLC cells and induces cell death via cell cycle arrest and apoptosis. CuD treatment or EGFR knockdown also suppressed the growth of gefitinib-resistant NSCLC cells. In addition, CuD overcame resistance by blocking EGF binding to EGFR in gefitinib-resistant NSCLC cells. In conclusion, we demonstrate that CuD overcomes gefitinib resistance by reducing the activation of EGFR-mediated survival in NSCLC and by inhibiting the combination of EGF and EGFR.

## Introduction

Lung cancer is currently the most common cause of death (1), and non-small cell lung cancer (NSCLC) accounts for a high proportion of the many pathological types (2). Approximately 15% of NSCLC patients have mutations in the tyrosine kinase domain of the epidermal growth factor receptor (EGFR) gene, and most of these patients respond to EGFR tyrosine kinase inhibitors (TKIs) (1, 3, 4). The EGFR family consists of several homologous members, including EGFR (ErbB1 or HER1), ErbB2 (HER2), and ErbB3 (HER3) (5, 6). These receptors contain an intracellular tyrosine kinase region and extracellular ligand binding sites (7). Ligand binding activates not only intrinsic protein kinase activity by EGFR dimerization but also the mitogen-activated protein kinase/extracellular signal-regulated kinase (MAPK/ERK) and phosphoinositide-3-kinase/protein kinase B (PI3K/AKT) signaling pathways to initiate the cytoplasmic signal transduction pathway. The activation of these pathways has been demonstrated to regulate cell proliferation and survival in many studies (8–10).

Gefitinib is a first-generation EGFR-TKI and a typical treatment recommended for NSCLC patients with activating EGFR mutations (11, 12). However, NSCLC patients who initially respond well to EGFR-TKIs eventually acquire resistance to gefitinib, and it is urgent that effective treatments to overcome gefitinib resistance are developed (13).

Naturally occurring dietary compounds have increased the interest in the prevention of various types of cancers (14–16), including lung cancer (17–20). Cucurbitacin is commonly found in the cucurbitaceae family and has been used in traditional medicine (21). Although cucurbitacin shows some toxicity, it is notable that the toxic dose of cucurbitacin is much greater than the active dose, which increases its potential as a treatment (22). Among various studies of the cucurbitaceae family, cucurbitacin D (CuD) has been shown to have anticancer effects in a variety of cancers, including breast cancer, cervical cancer, prostate cancer, and gastric cancer (23–27). Previous studies have shown that CuD is likely to be used as an agent to inhibit cancer progression and that the compound could potentially improve chemotherapy in the future, but there have been no studies in lung cancer.

Our studies demonstrate that CuD overcomes gefitinib resistance by blocking EGF binding to induce EGFR-mediated signaling and cell death, which suggests that CuD treatment could be useful for treating gefitinib-resistant lung cancer.

## Materials and Methods

### Cell Culture and Generation of Gefitinib-Resistant HCC827 Cells

The non-transformed immortalized human epithelial lung cell line NL20 was obtained from ATCC. The human NSCLC cell line HCC827 was obtained from the Korea Cell Line Bank (KCLB, Seoul, South Korea); HCC827 cells have a mutation in the EGFR tyrosine kinase domain (E746-A750 deletion) (28). The HCC827GR cell line has been used as a gefitinib-resistant cell model. We exposed HCC827 cells to increasing concentrations of gefitinib (Gef; Sigma, St. Louis, MO, United States) according to a previously described method (29). Finally, HCC827 cells with stable gefitinib resistance were generated and named HCC827GR. The HCC827GR cell line was isolated and independently confirmed to be resistant to gefitinib. The cells were grown in RPMI-1640 (Welgene, Daegu, Korea) supplemented with 10% heat-inactivated fetal bovine serum (Welgene) and 1% antibiotics (Ab; Welgene) at 37°C in a 5% CO_2_ humidified incubator.

### Cell Viability Assay

Cell viability was assessed using an 3-(4,5-dimethylthiazol-2-yl)-2-5-diphenyltetrazolium bromide (MTT) assay. Briefly, NL20, HCC827, and HCC827GR cells (1 × 10^4^ cells/well) were seeded into 96-well plates and incubated overnight in a 5% CO_2_ atmosphere at 37°C. The media was then removed, and the cells were treated with EGF (50 ng/ml, Sigma) and/or gefitinib and/or CuD (Extrasynthese, Genay Cedex, France) and incubated for another 24, 48, and 72 h. Next, 100 μl of MTT solution (1 mg/ml, Sigma) was added to each well, and the plate was incubated for another 4 h at 37°C. The formazan crystals formed were dissolved in dimethyl sulfoxide (200 μl per well) with constant shaking for 5 min. Optical density was determined at 560 nm using an ELISA reader (Versa Max, Molecular Devices, Sunnyvale, CA, United States). This assay was conducted in triplicate.

### Cell Migration Assay

For wound-healing assays, cells were grown on a six-well plate until 85% confluence, and the monolayers were scratched with a pipette tip. Cell migration was recorded at 0 and 3 days after the wound scratch. Migration rates were counted with ImageJ software (version 1.42q; National Institutes of Health, Bethesda, MD, United States). Cell migration was analyzed by taking images using a camera connected to a light microscope (Olympus, Tokyo, Japan). The experiment was repeated independently three times.

### Colony Formation Assay

Cells were seeded in six-well plates at a concentration of 1,000 cells per well, allowed to attach overnight, and treated with 0.01 μM Gef or CuD or 0.1 μM Gef or CuD. Culture medium and chemicals were replaced every 72 h. After 10–14 days, the cells were stained with a solution mixture of 0.5% crystal violet and 6% glutaraldehyde (Sigma). Colonies were counted with ImageJ software (version 1.42q; National Institutes of Health, Bethesda, MD, United States). Colony formation was analyzed by taking images using a camera connected to a light microscope.

### Solid-Phase Binding Assay

A 96-well microplate (Thermo Fisher Scientific, Massachusetts, United States) was coated with 100 μl of phosphate-buffered saline (PBS) containing 100 ng/ml human-EGFR, ErbB2, or ErbB3 ECD/Fc chimera (R&D Systems, Minneapolis, USA). The plate was incubated overnight at 4°C. After three washes with 200 μl of PBS containing 0.05% (*v*/*v*) Tween 20, the plate was blocked by adding 200 μl of PBS with 2% (*w*/*v*) bovine serum albumin (BSA) and incubated for 2 h at room temperature. The plate was washed three times, and 100 μl of diluted standards (biotinylated EGF, R&D systems) or CuD (with 5 ng/ml biotinylated EGF) in PBS was added. After 2.5 h of incubation at room temperature, the plate was washed three times, and 100 μl of streptavidin horseradish peroxidase (R&D Systems) diluted 1:250 in blocking buffer was added. Finally, tetramethylbenzidine substrate solution (BD Biosciences, San Diego, United States) was added to the plates, and after a 1-h incubation in the dark, a 1 M H_3_PO_4_ solution was added to stop the reaction. After incubation for 1–3 h at room temperature, 50 μl of stop solution (1 M H_3_PO_4_) was added to each well. The signal was measured at 450 nm using an ELISA plate reader (Versa Max, Molecular Devices). The method was performed as described previously (30). The assays were performed in triplicate. The data were fit to the equation for log (inhibitor) vs. response using PRISM software (GraphPad Software Inc., La Jolla, CA, United States). The significance of the differences between the IC_50_ values in the absence and presence of EGF-EGFR/ErbB2/ErbB3 was based on the *p* value assigned to those differences by PRISM software.

### Immunofluorescence Assay

For immunofluorescence, cells were fixed with 3–4% paraformaldehyde in 0.1 M PBS for 15 min, permeabilized with 0.25% Triton X-100 for 10 min and blocked with 1% BSA for 1 h. Following rinsing with PBS, the coverslips with adherent cells were used for immunofluorescence staining. In every group, the cells were incubated with anti-p-EGFR (Y1068) primary antibody (1:100; Cell Signaling Technology, Danvers, MA, United States) overnight at 4°C. Subsequently, the cells were incubated with an Alexa488-conjugated secondary antibody (1:500; Invitrogen, Eugene, Oregon, United States) for 1 h at room temperature. After washing, the coverslips were mounted using fluorescent mounting medium with 4,6-diamidino-2-phenylindole (Sigma, EMD Millipore, Billerica, MA, USA). Images were obtained with an Olympus FV10i Self-Contained Confocal Laser System (Fluoview1000, Olympus, Tokyo, Japan). The objective was 40×, and the scale bars on the image indicate 20 μm.

### Western Blot Analysis

Cells were harvested, lysed with cell lysis buffer (50 mM Tris–Cl, pH 7.4, 1% NP-40, 0.25% sodium deoxycholate, 0.1% sodium dodecyl sulfate, 150 mM NaCl, 1 mM ethylenediaminetetraacetic acid, and protease inhibitor) on ice for 30 min and centrifuged at 13,000 rpm and 4°C for 20 min. The lysates were separated by centrifugation at 13,000 rpm for 20 min at 4°C. The supernatants were stored at −70°C until use. Protein concentrations were quantified using a Bio-Rad Bradford protein assay (Bio-Rad, Hercules, CA, United States). Next, total protein samples were electrophoresed using 8–15% reducing sodium dodecyl sulfate polyacrylamide gels and transferred to nitrocellulose membranes (Protran nitrocellulose membrane, Whatman, United Kingdom). After blocking with 0.1% Tween-20 in PBS containing 1% skim milk and 1% BSA for 1 h, the membranes were incubated overnight at 4°C with the indicated primary antibodies. After washing with 1× PBS with Tween®, the membranes were incubated with diluted enzyme-linked secondary antibodies. After washing with 1× PBS with Tween®, the protein bands were detected using an EZ-western chemiluminescent detection kit and visualized by exposing the membranes to X-ray films. Each protein was blotted with the appropriate antibodies as follows: anti-EKR1/2, protein kinase B (AKT), cdc2, cdc25c, p-EKR1/2, p-AKT, p-cdc2 (Tyr15), p-cdc25c (Ser216), and cyclin B1 antibodies were purchased from Santa Cruz Biotechnology (Santa Cruz, CA, United States); anti-EGFR, ErbB2, ErbB3, c-MET, p-EGFR (Y1068), p-ErbB2, p-ErbB3, p-c-MET, cleaved poly(ADP-ribose) polymerase (PARP), and glyceraldehyde 3-phosphate dehydrogenase (GAPDH) antibodies were obtained from Cell Signaling Technology (Danvers, MA, United States).

### Cell Cycle Analysis

Flow cytometry was used to analyze the cell cycle. In this experiment, ~70% confluent cells were seeded into six-well plates and treated with CuD or gefitinib for 24 h. Trypsinized cells were washed twice with ice-cold 1× PBS. The cell pellets were resuspended in ice-cold 1× PBS and fixed in 95% ethanol at 4°C. The cells were washed twice with ice-cold 1× PBS, suspended in 1× PBS, stained with a propidium iodide staining solution (BD Biosciences, San Jose, CA, United States), and analyzed by a BD FACSCalibur Flow Cytometer (BD Biosciences) following the manufacturer's instructions.

### Apoptosis Analysis

Flow cytometry was used to analyze cell apoptosis. In this experiment, ~60% confluent cells were seeded into six-well plates and treated with CuD or gefitinib for 72 h. The apoptosis assay was performed with an Annexin V-FITC/PI double staining apoptosis detection kit (BD Biosciences) and a BD FACSCalibur Flow Cytometer following the manufacturer's instructions.

### *In vitro* Transfection With siRNAs

Small interfering RNAs (siRNAs) targeting EGFR were synthesized by Santa Cruz Biotechnology (Santa Cruz, CA, United States). In addition, a non-specific scrambled siRNA was purchased from Santa Cruz Biotechnology (Santa Cruz, CA, United States) and used as a control. siRNA transfection was performed according to the manufacturer's instructions. Briefly, 24 h before transfection, six-well plates were seeded with 1 × 10^4^ cells per well in 2 ml of culture medium. The cells were transfected with EGFR or scrambled siRNA with 1 ml of Lipofectamine iMAX reagent (Invitrogen, Carlsbad, CA, United States) according to the manufacturer's protocol. Twenty-four hours later, the cells were treated with CuD. For the MTT assay, the cells from the six-well plate were reseeded in a 96-well plate 24 h after transfection.

### Statistical Analysis

All experimental results are expressed as the mean ± standard deviation (SD) or the mean ± SEM of at least three separate analyses. Student's *t* test was used for single variable comparisons, and a *P* < 0.05 was considered statistically significant. All experiments were performed at least three times. Statistical analyses were performed using Prism software (GraphPad Software Inc., La Jolla, CA, United States).

## Results

### Cucurbitacin D Suppresses the Phosphorylation of EGFR in Gefitinib-Resistant NSCLC Cells

**Figure 1:**
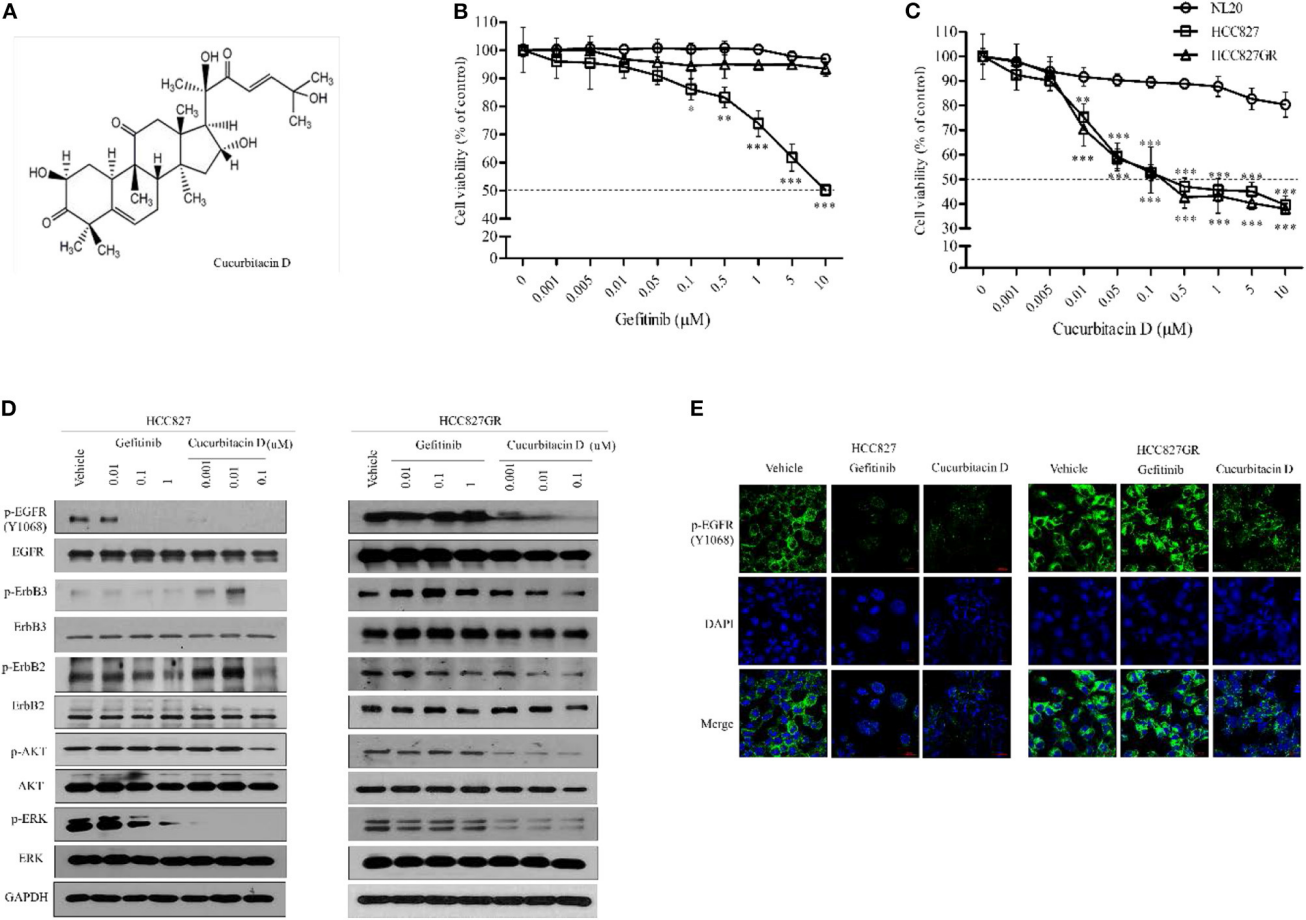
Cucurbitacin D suppresses the EGFR signaling pathway. **(A)** Chemical structure of cucurbitacin D. **(B,C)** The cytotoxicity of gefitinib **(B)** and cucurbitacin D **(C)** in cells was measured by 3-(4,5-dimethylthiazol-2-yl)-2–5-diphenyltetrazolium bromide (MTT) assay. NL20, HCC827, and HCC827GR cells were treated with various concentrations of gefitinib or cucurbitacin D for 24 h. **(D)** Cucurbitacin D and gefitinib inhibit the activity of the EGFR signaling pathway. HCC827 (left panel) and HCC827GR (right panel) cells were treated with cucurbitacin D or gefitinib for 24 h, as indicated. Western blotting was conducted to detect the target proteins. The values shown above the blots are an analysis of the blots normalized to glyceraldehyde 3-phosphate dehydrogenase (GAPDH). **(E)** Immunofluorescence (IF) analysis of intracellular p-EGFR (Y1068) accumulation in HCC827 and HCC827GR cells treated under the indicated conditions for 24 h. The cells were immunostained with a p-EGFR (Y1068) antibody (green) and counterstained with 4,6-diamidino-2-phenylindole (DAPI) (blue). Bar = 20 μm. Fluorescence microscopy images for all cell lines. *P < 0.05, **P < 0.01, and ***P < 0.001 compared with the non-transformed immortalized human epithelial lung cell line NL20. The data are presented as the mean ± SEM.

First, we performed an MTT assay to determine the cytotoxicity of CuD (Figure 1A) and gefitinib in NSCLC cells (HCC827 and HCC827GR) and non-transformed immortalized human epithelial lung cells (NL20) (Figure S1). To explore the effects of CuD on acquired gefitinib-resistant cells, we generated HCC827 cells with acquired gefitinib resistance (HCC827GR) from their parental cells (HCC827) by continuous exposure to gefitinib and CuD at 0–10 μM for 24 h. As shown in Figure 1B, gefitinib exhibited less cytotoxicity in established HCC827GR cells than in HCC827 cells at a concentration of 0.1 μM. However, CuD exhibited slightly higher cytotoxicity in established HCC827GR cells than in HCC827 cells at a concentration of 0.01 μM, but it did not affect NL20 cells (Figure 1C). In particular, the CuD results were clearly observed in HCC827GR cells as well as in HCC827GR cells, suggesting that CuD may be able to overcome acquired gefitinib-resistant cells.

Next, to explore the fundamental mechanism by which CuD inhibited cell growth in NSCLC, the protein expression of EGFR in HCC827 and HCC827GR cell lines was analyzed, as well as its effect. As expected, treatment with gefitinib inhibited the subsequent AKT and ERK activation in gefitinib-sensitive HCC827 cells to phosphorylate EGFR, ErbB2, and ErbB3, but this effect did not occur in gefitinib-resistant HCC827GR cells (Figure 1D). Moreover, the phosphorylation of EGFR, ErbB2, ErbB3, AKT, and ERK proteins was decreased in all tested cells (HCC827 and HCC827GR), and CuD specifically targeted EGFR in HCC827GR cells (Figure 1D). According to previous reports, protein expression of EGFR, ErbB2, and ErbB3 were observed to be high in HCC827 cells while significantly low in NL20 cells (31, 32). In NL20 cells, both CuD and gefitinib had little effect on the phosphorylation rates of EGFR, ErbB2, ErbB3, AKT, and ERK proteins (Figure S2). In previous studies, MET amplification was demonstrated to activate EGFR-TKI mutations in the HCC827 cell line with gefitinib resistance (33), and we found MET amplification in HCC827GR cells (Figure S3). However, CuD was observed to slightly suppress MET activity and was expected to have a greater effect on the phosphorylation of EGFR and overcome gefitinib resistance, as shown in Figure S3.

We also measured punctate fluorescence by immunofluorescent staining in CuD- or gefitinib-treated HCC827 and HCC827GR cells (Figure 1E). In HCC827GR cells, CuD induced more punctate fluorescence than in HCC827 cells, which showed only slight fluorescence. The results showed EGFR phosphorylation in HCC827GR cells after 24 h of CuD or gefitinib treatment, with the same results as those in Figure 1D.

**Figure 2:**
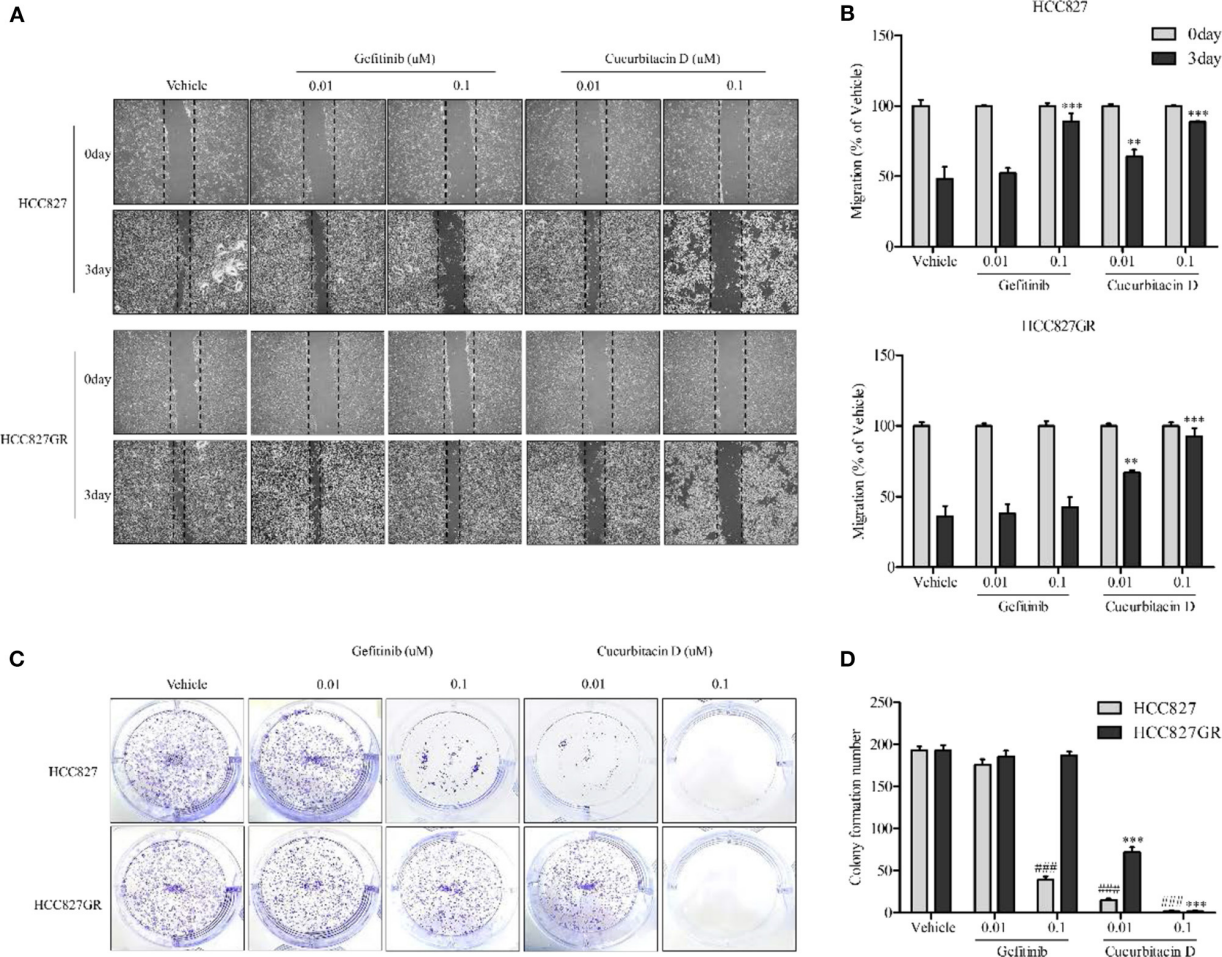
Cucurbitacin D inhibits cell migration and growth. **(A,B)** Wound healing assay. Cell migration was assessed by healing of the scratch. The area of the wound was measured at two time points (days 0 and 3) in every group, and the percent reduction of the initial scratch area was compared. The arrows indicate the boundary lines of the scratch. **(A)** Representative images of all panel results for HCC827 and HCC827GR cells (×40). **(B)** The bar graphs represent the quantification of Western blot data for HCC827 (upper panel) and HCC827GR (lower panel) cells. **P < 0.01 and ***P < 0.001 compared to vehicle (3 days). **(C,D)** Colony formation assay. HCC827 and HCC827GR cells were seeded in six-well plates and treated with gefitinib (0.01 and 0.1 μM) or cucurbitacin D (0.01 and 0.1 μM) for 10–14 days before being stained **(C)**, and the number of colonies was quantified **(D)**. ^###^*P* < 0.001 compared to vehicle HCC827 cells. ***P < 0.001 compared to vehicle HCC827GR cells. All data are presented as the mean ± SD.

While EGFR signaling activation increased proliferation, migration rate, and cell survival as previously reported (34, 35), CuD did not affect NSCLC migration. The effect of CuD on the migration rate was measured. HCC827 and HCC827GR cells were treated with 0.01 μM CuD and/or gefitinib and 0.1 μM CuD or gefitinib for 3 days (Figures 2A,B). CuD decreased the number of migrated cells into the scratch region in HCC827 and HCC827GR cells treated for 3 days. Likewise, CuD and/or gefitinib suppressed the growth of HCC827 and HCC827GR cells in a 14-day growth inhibition colony assay. It was found that 0.1 μM CuD caused a 98% reduction in the number of HCC827 and HCC827GR cell colonies. In contrast, 0.1 μM gefitinib caused an 80 and 1% reduction in the number of HCC827 and HCC827GR cell colonies, respectively (Figures 2C,D). Surprisingly, 0.1 μM CuD almost completely inhibited cell growth and migration in all tested cells. Together, these findings suggest that CuD treatment suppresses EGFR phosphorylation, cell migration, and cell growth in both gefitinib-sensitive HCC827 cells and gefitinib-resistant HCC827GR cells.

### Cucurbitacin D Induces Cell Death via Cell Cycle Arrest and Apoptosis in Gefitinib-Resistant NSCLC Cells

**Figure 3:**
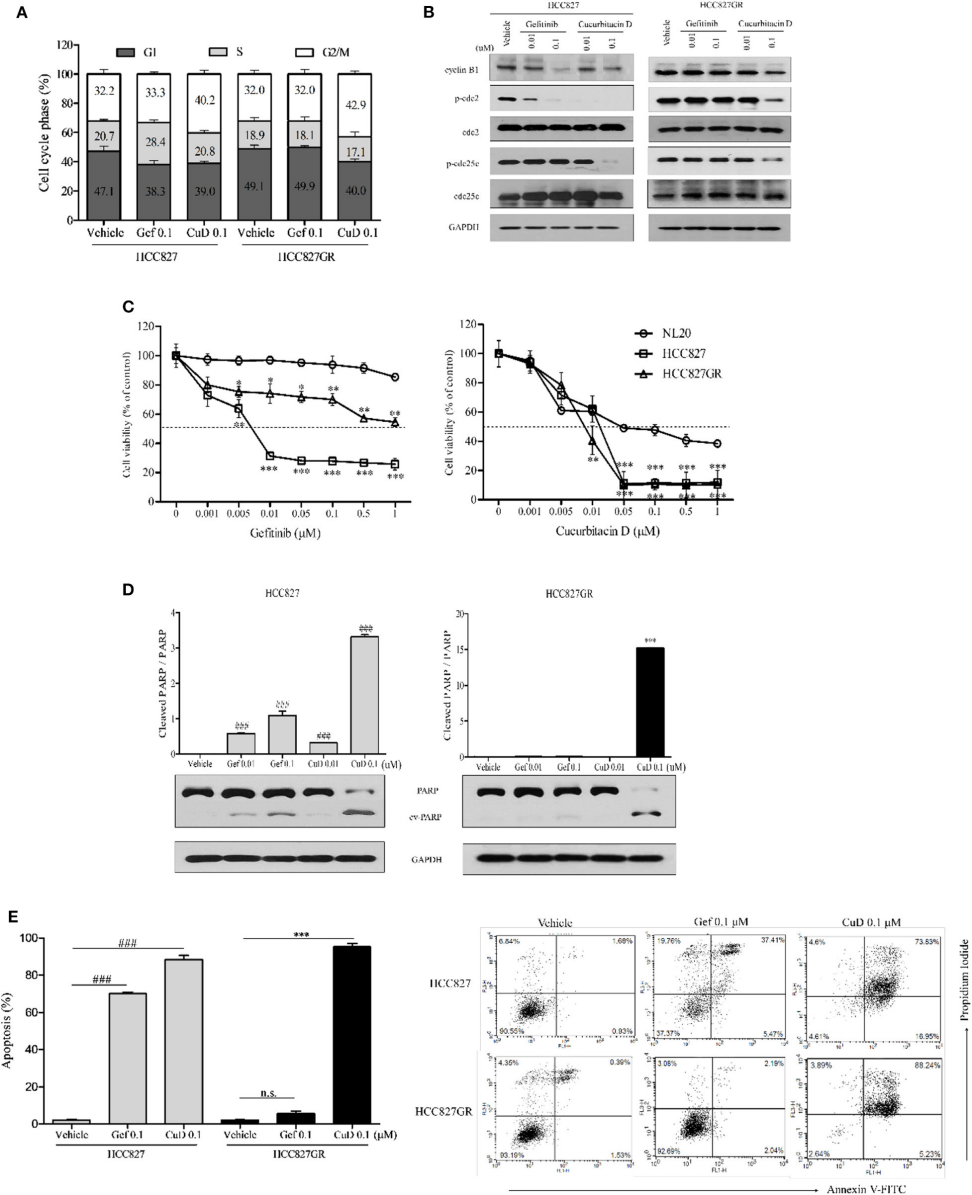
Cucurbitacin D induces cell cycle arrest and apoptosis. **(A)** PI staining analysis. Treatment with cucurbitacin D or gefitinib determined cell cycle arrest in HCC827 and HCC827GR cells. The data are presented as the mean ± SD. **(B)** Effect of cucurbitacin D or gefitinib on the levels of G2/M phase cell cycle regulatory proteins. HCC827 (left panel) and HCC827GR (right panel) cells were treated with cucurbitacin D or gefitinib for 24 h, as indicated. Western blotting was conducted to detect the target proteins. The values shown above the blots are an analysis of the blots normalized to glyceraldehyde 3-phosphate dehydrogenase (GAPDH). **(C)** The cytotoxicity of gefitinib and cucurbitacin D was measured in cells by 3-(4,5-dimethylthiazol-2-yl)-2-5-diphenyltetrazolium bromide (MTT) assay. HCC827 and HCC827GR cells were treated with gefitinib (left panel) and cucurbitacin D (right panel) for 72 h. The data are presented as the mean ± SEM. *P < 0.05, **P < 0.01, and ***P < 0.001 compared with the non-transformed immortalized human epithelial lung cell line NL20. **(D)** Effect of cucurbitacin D or gefitinib on the levels of apoptosis regulatory-related proteins (PARP and p-PARP). HCC827 (left panel) and HCC827GR (right panel) cells were treated with cucurbitacin D or gefitinib for 72 h, as indicated. Western blotting was conducted to detect the target proteins. The values shown above the blots are an analysis of the blots normalized to GAPDH. The data are presented as the mean ± SD. ^###^*P* < 0.001 and ***P < 0.001 compared with vehicle HCC827 cells and vehicle HCC827GR cells, respectively. **(E)** Annexin V/PI staining analysis. Treatment with cucurbitacin D or gefitinib affected apoptosis in HCC827 and HCC827GR cells. The numbers represent percentage of cells in the appropriate quadrant. Left lower quadrant, viable cells; right lower quadrant, early apoptotic cells; right upper quadrant, late apoptotic cells; ^###^*P* < 0.001 and ***P < 0.001 compared with vehicle HCC827 cells and vehicle HCC827GR cells, respectively. The data are presented as the mean ± SD. CuD, cucurbitacin D; Gef, gefitinib; n.s., no significance.

Interestingly, the inhibitory effect of CuD on cell growth and migration was slightly greater in HCC827GR cells than in HCC827 cells (Figure 2). As shown in Figure 3A, CuD was able to increase G2/M phase arrest in HCC827GR cells, but gefitinib did not cause this effect at all. Similarly, we examined the effect of CuD on apoptosis and proteins associated with apoptosis and cell cycle arrest. Western blot results showed that treatment with CuD for 24 h reduced the phosphorylation of cdc2 and cdc25c and suppressed cyclin B1 expression upon inducing G2/M phase arrest in both HCC827 and HCC827GR cells (Figure 3B). We next performed an MTT assay to investigate the 72-h treatment effect of CuD on NSCLC cells. The IC_50_ of CuD was 0.05 μM in NL20 cells (Figure 3C). Treatment with CuD also increased the expression of cleaved PARP, indicating the potential of CuD for inducing death signaling in gefitinib-resistant HCC827GR cells (Figure 3D). In addition, we performed Annexin-V staining and flow cytometry analyses to prove that CuD could induce apoptosis in HCC827 and HCC827GR cells. As shown in Figure 3E, when treated with 0.1 μM gefitinib, the apoptosis rate increased 70.14 ± 1.48% in HCC827 cells, as expected, but not in HCC827GR cells. Moreover, 0.1 μM CuD increased cell apoptosis to 88.39 ± 4.77 and 95.28 ± 3.63% in HCC827 and HCC827GR cells, respectively. Therefore, CuD was confirmed to overcome resistance to gefitinib by inducing apoptosis and enhancing cell cycle arrest.

### Cucurbitacin D or EGFR Knockdown Inhibits Cell Growth in Gefitinib-Resistant NSCLC Cells

**Figure 4:**
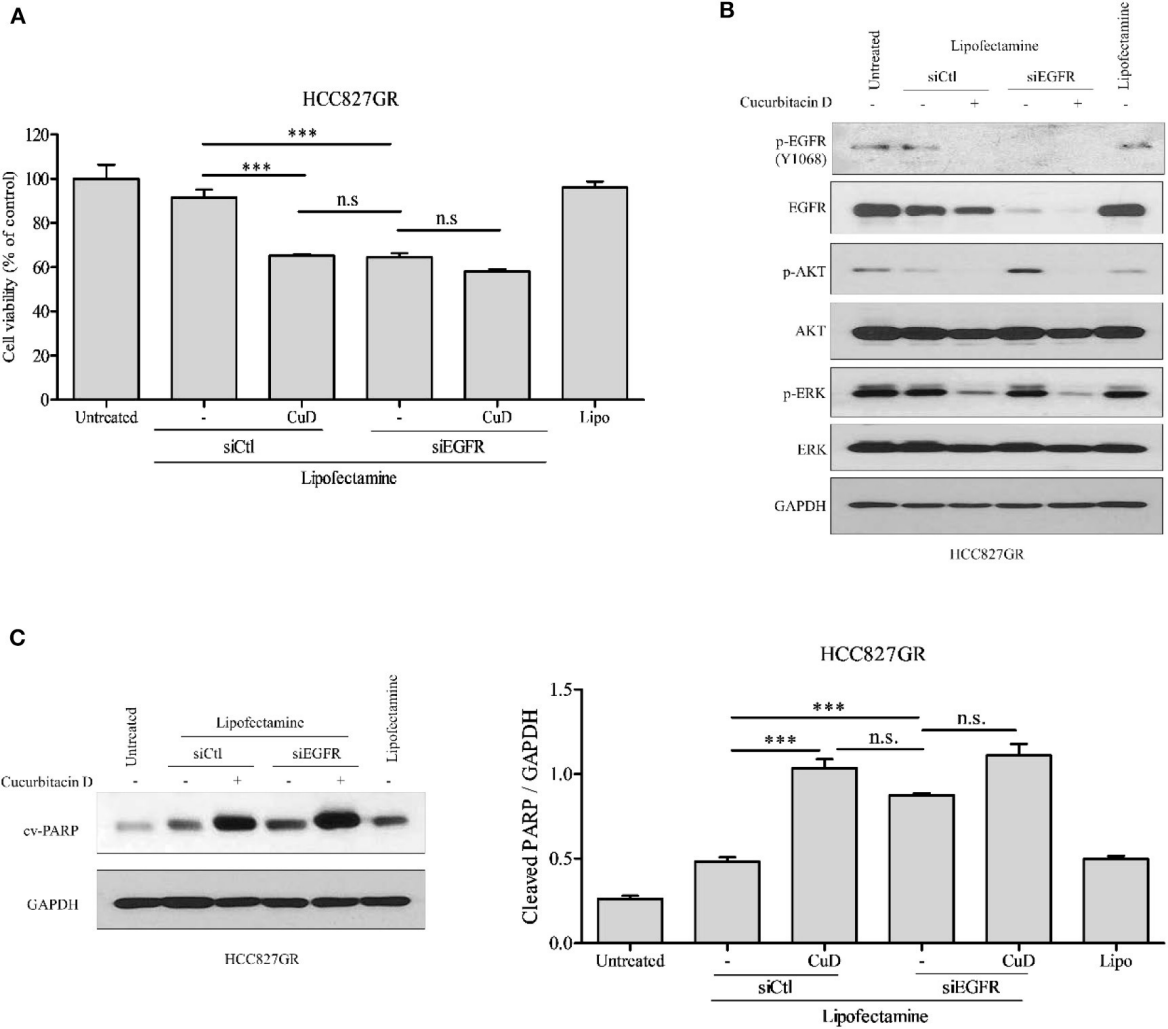
EGFR knockdown or cucurbitacin D treatment inhibits cell growth. **(A)** HCC827GR cells were reseeded in a 96-well plate after transfection with 10 μM control small interfering RNA (siRNA) or 10 μM EGFR siRNA for 24 h. After days of treatment with 0.1 μM cucurbitacin D, an 3-(4,5-dimethylthiazol-2-yl)-2-5-diphenyltetrazolium bromide (MTT) assay was performed. **(B,C)** Effect of EGFR knockdown or cucurbitacin D treatment on the levels of cell death regulatory-related proteins. After transfection for 24 h, we reseeded the cells for Western blotting. Cells were treated with cucurbitacin D for 24 h, and Western blotting was conducted to detect EGFR signaling **(B)** and cleaved poly(ADP-ribose) polymerase (PARP) **(C)** protein levels. The values shown above the blots are an analysis of the blots normalized to glyceraldehyde 3-phosphate dehydrogenase (GAPDH). ***P < 0.001 compared with control siRNA cells. All data are presented as the mean ± SD. CuD, cucurbitacin D; n.s., no significance; siCtl, control siRNA.

Previous studies have shown that CuD effectively inhibits the phosphorylation of EGFR in two experimental cell lines (HCC827 and HCC827GR) (Figures 1D,E). This study aimed to determine whether CuD could overcome gefitinib resistance, so we used EGFR knockdown (siEGFR) siRNA in HCC827GR cells and treated them with CuD. CuD and siEGFR powerfully reduced the viability of HCC827GR cells by ~65% (Figure 4A). Western blot results showed that CuD and siEGFR similarly inhibited EGFR phosphorylation and similarly increased cleaved-PARP in HCC827GR cells (Figures 4B,C). CuD together with siEGFR was observed to slightly but not significantly increase cleaved-PARP levels compared to siEGFR alone (Figure 4C). This result likely occurred because CuD affects the EGFR family, ErbB2 and ErbB3, as shown in Figure 1. Along with the EGFR phosphorylation results, CuD also strongly inhibited EGFR downstream pathways, including AKT and ERK (Figure 4B). These results provide clear evidence for CuD inhibition of the cell growth rate in gefitinib-resistant NSCLC cells via EGFR.

### Cucurbitacin D Overcomes Resistance by Blocking EGF Binding to EGFR in Gefitinib-Resistant NSCLC Cells

**Figure 5:**
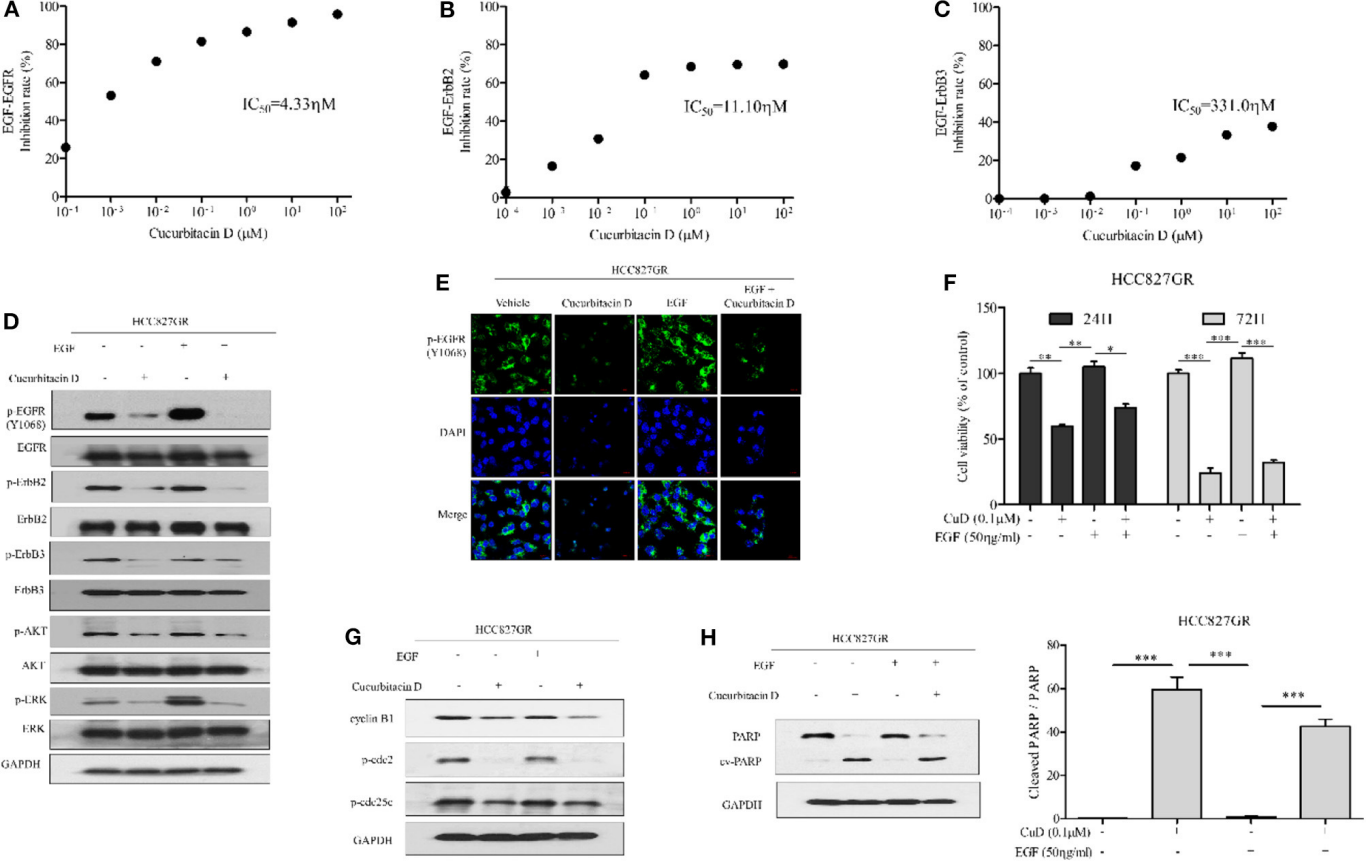
Cucurbitacin D inhibits EGF binding to EGFR and EGF-induced signaling. **(A–C)** Cucurbitacin D and biotin-EGF were added to a 96-well plate coated with recombinant human EGFR **(A)**, ErbB2 **(B)**, or ErbB3 **(C)**. **(D)** Cucurbitacin D inhibition of EGF-induced EGFR signaling was examined by Western blot. HCC827GR cells were pretreated with 0.1 μM CuD for 24 h as indicated and stimulated with EGF (50 ng/ml) for 2 h. The values shown above the blots are an analysis of the blots normalized to glyceraldehyde 3-phosphate dehydrogenase (GAPDH). **(E)** Immunofluorescence (IF) analysis of intracellular p-EGFR (Y1068) accumulation in the presence of EGF and cucurbitacin D. HCC827GR cells were immunostained with a p-EGFR (Y1068) antibody (green) and counterstained with 4,6-diamidino-2-phenylindole (DAPI) (blue). Bar = 20 μm. Fluorescence microscopy images of the cell lines. **(F)** Cell viability in the presence of EGF and cucurbitacin D was measured by 3-(4,5-dimethylthiazol-2-yl)-2-5-diphenyltetrazolium bromide (MTT) assay for 24 and 72 h. *P < 0.05, **P < 0.01, and ***P < 0.001. The data are presented as the mean ± SD. **(G,H)** Cucurbitacin D inhibition of EGF-induced cell cycle **(G)** and death **(H)** regulatory proteins was examined by Western blot. The values shown above the blots are an analysis of the blots normalized to GAPDH. ***P < 0.001. The data are presented as the mean ± SD. CuD, cucurbitacin D.

Based on the results of the effects of CuD on EGFR, ErbB2, and ErbB3 shown in Figure 1D, we proposed that CuD interacts strongly with these receptors. The EGFR family (EGFR; HER1; ErbB1, HER2; ErbB2 and HER3; ErbB3) consists of tyrosine kinases with different ligand specificity, and among these ligands, EGF works by binding common elements (36). We also focused on whether CuD inhibited EGF-dependent EGFR phosphorylation and downstream signaling. In the EGF-EGFR interaction analysis, CuD directly inhibited the interaction between EGF and EGFR (IC_50_ = 4.33 nM) (Figure 5A). CuD also inhibited the interaction between EGF and ErbB2 (IC_50_ = 11.10 nM) or ErbB3 (IC_50_ = 331.0 nM) but not as directly as the interaction between EGF and EGFR (Figures 5B,C). We next examined the effect of CuD on EGF-dependent cell migration and cell growth signaling. CuD inhibited EGF-induced intracellular EGFR, ErbB2, and ErbB3 signaling when HCC827GR cells were pretreated with 0.1 μM CuD and then stimulated with EGF (50 ng/ml) for 2 h (Figure 5D). CuD decreased EGF-dependent EGFR, ErbB2, and ErbB3 phosphorylation, resulting in a reduction in ERK and AKT phosphorylation. Western blot results confirmed that CuD obviously inhibited EGF-dependent EGFR, and the same results were also found through immunofluorescent staining (Figure 5E). As a result, CuD visibly inhibited intracellular p-EGFR accumulation in HCC827GR cells stimulated with EGF. Furthermore, cell viability was measured in HCC827GR cells stimulated by EGF (Figure 5F), and CuD reduced the phosphorylation of ERK and AKT, related to cell migration and growth (Figure 5D). As expected, in HCC827GR cells stimulated by EGF, CuD significantly decreased cell viability, and apoptosis and cell cycle arrest were further observed through Western blotting. Figure 5G shows that in HCC827GR cells stimulated by EGF, CuD inhibited the phosphorylation of cdc2 and cdc25c and similarly inhibited cyclin B1 to induce G2/M phase arrest. Similarly, CuD was found to induce apoptosis by increasing cleaved PARP levels in HCC827GR cells stimulated by EGF, but not higher than the CuD alone (Figure 5H). Moreover, further investigation through immunofluorescent staining and cell viability assays showed that gefitinib had no effect on HCC827GR cells stimulated by EGF (Figures S4A,B). In conclusion, we identified that CuD directly inhibits the EGF–EGFR interaction and competitively overcomes resistance in gefitinib-resistant NSCLC cells stimulated by EGF.

## Discussion

The EGFR family consists of membrane tyrosine kinases with different ligand specificities (36, 37). The binding to ligands induces the activation of homo- or heterodimerization and kinase domains that initiate cascades of cytoplasm and nuclear amplification pathways (including the mitogen-activated protein kinase and AKT pathways), leading to gene activation and cell proliferation (38). Gefitinib, recently found to be an EGFR- TKIs, is one of the most common treatments for NSCLC involving EGFR mutations; however, almost all NSCLC patients become resistant (13). Thus, it is urgent that an effective drug to overcome gefitinib resistance is developed. We identified how CuD affected the EGFR family (EGFR, ErbB2, and ErbB3) and observed that it overcomes gefitinib resistance in NSCLC cells.

First, CuD inhibited phosphorylation of the EGFR family in HCC827 cells and gefitinib-resistant HCC827 cells (HCC827GR), but of these EGFR family members, CuD had the greatest effect on EGFR phosphorylation. Gefitinib, however, showed this tendency in only HCC827 cells, and it was completely ineffective in HCC827GR cells. Furthermore, immunofluorescence assays revealed that CuD significantly inhibited the expression of EGFR in the two experimental cell lines; in contrast, gefitinib did not inhibit EGFR expression in HCC827GR cells, confirming the results of previous Western blot assays. CuD also significantly inhibited the phosphorylation of ERK and AKT, the downstream EGFR signaling pathway related to cell migration and growth, in HCC827 and HCC827GR cells. However, gefitinib did not have this effect in HCC827GR cells. In addition, we analyzed cell migration and survival through migration and colony formation assays, respectively; CuD appeared to cause equal reductions in all experimental cells, and gefitinib had no effect on HCC827GR cells.

According to a previous study, the presence of mesenchymal–epidermal transition (MET) receptor amplification was analyzed as a second mechanism of gefitinib resistance; 20% of EGFR T790M-resistant patients among a number of lung cancer patients were observed to have increased MET receptor levels (39–41). Although it was confirmed that MET receptor levels were higher in HCC827GR cells than in HCC827 cells, CuD unfortunately had no effect on MET receptor amplification.

Deregulation of the cell cycle is one of the manifestations of human cancer (42, 43). In particular, CuD strongly induced G2/M phase arrest and apoptosis in HCC827 and HCC827GR cells, but interestingly, the induction of apoptosis in HCC827GR cells was increased significantly. Further monitoring of CuD responses to EGFR using siRNA showed similar responses to EGFR knockdown and CuD treatment alone in HCC827GR cells. These results again indicated that EGFR is an important target in overcoming gefitinib resistance. EGF activation of EGFR-mediated signaling is a main driver of proliferation, migration, and cell survival (34, 35). While the CuD concentration required to suppress EGF/ErbB2 and EGF/ErbB3 binding was higher than that required to suppress EGF/EGFR binding, CuD was found to inhibit EGF binding as a whole. In this study, we found that CuD significantly induced cell death by blocking EGF binding to EGFR in HCC827GR cells. In addition, we found that CuD clearly inhibits the interaction between EGF and EGFR.

In conclusion, our data indicate that CuD overcomes gefitinib resistance by interrupting the interaction between EGF and EGFR and thereby regulating apoptosis. Clearly, effective treatment is needed for NSCLC patients with gefitinib resistance, and our results suggest CuD as a reasonable compound to support the treatment and development of new anticancer drugs targeting EGFR.

## Data Availability Statement

All data and materials are described within the article. The corresponding authors will provide data and materials upon request.

## Author Contributions

SH carried out the experiment and drafted the manuscript. SH, JMK, and YL revised the data and manuscript and assisted in the research work. SH, SL, and JHK guided the research and revised and submitted the manuscript. CC and S-GK supervised the research. All the authors read and approved the final manuscript.

### Conflict of Interest

The authors declare that the research was conducted in the absence of any commercial or financial relationships that could be construed as a potential conflict of interest.

## Abbreviations

CuD: cucurbitacin D
EGFR: epidermal growth factor receptor
TKIs: tyrosine kinase inhibitors
NSCLC: non-small cell lung cancer
MET: mesenchymal–epidermal transition.
