# Supplementary material for: Cucurbitacin D Overcomes Gefitinib Resistance by Blocking EGF Binding to EGFR and Inducing Cell Death in NSCLCs
Source: Front Oncol. 2020 Feb 18;10:62. doi: 10.3389/fonc.2020.00062 (PMC7041627; doi:10.3389/fonc.2020.00062)
Supplement: Supplementary file 1 [file Data_Sheet_1.pdf]

Supplementary Material

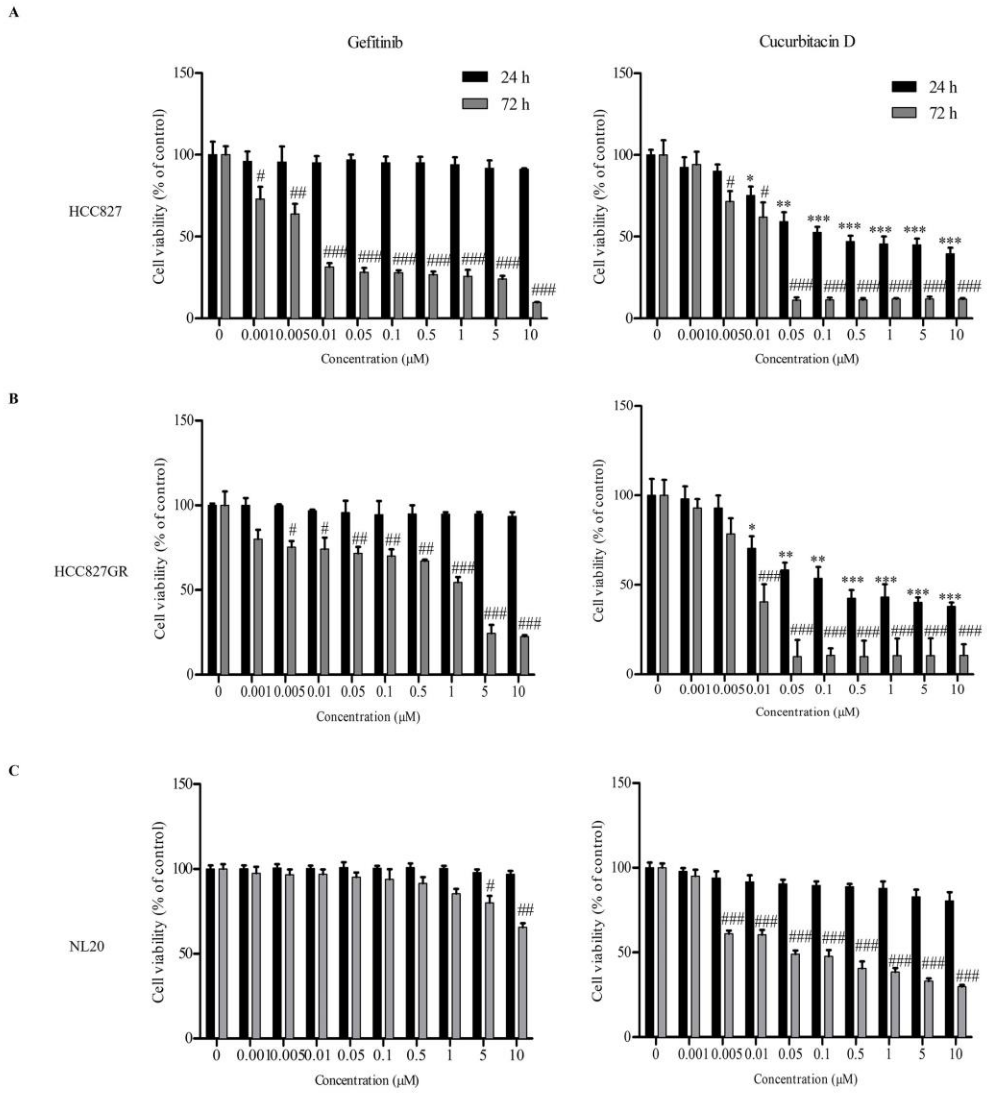

Figure S1. Measurement of cell viability after cucurbitacin D and gefitinib treatment. (A-C) Cell viability in the presence of gefitinib and cucurbitacin D for 24 and 72 hours was measured by MTT assay in HCC827 (A), HCC827GR (B) and NL20 (C) cells. *P < 0.05, **P < 0.01 and ***P < 0.001 compared with non-treated cells for 24 hours. #P < 0.05, ##P < 0.01 and ###P < 0.001 compared with non-treated cells for 72 hours. All data are presented as the mean ± S.D.

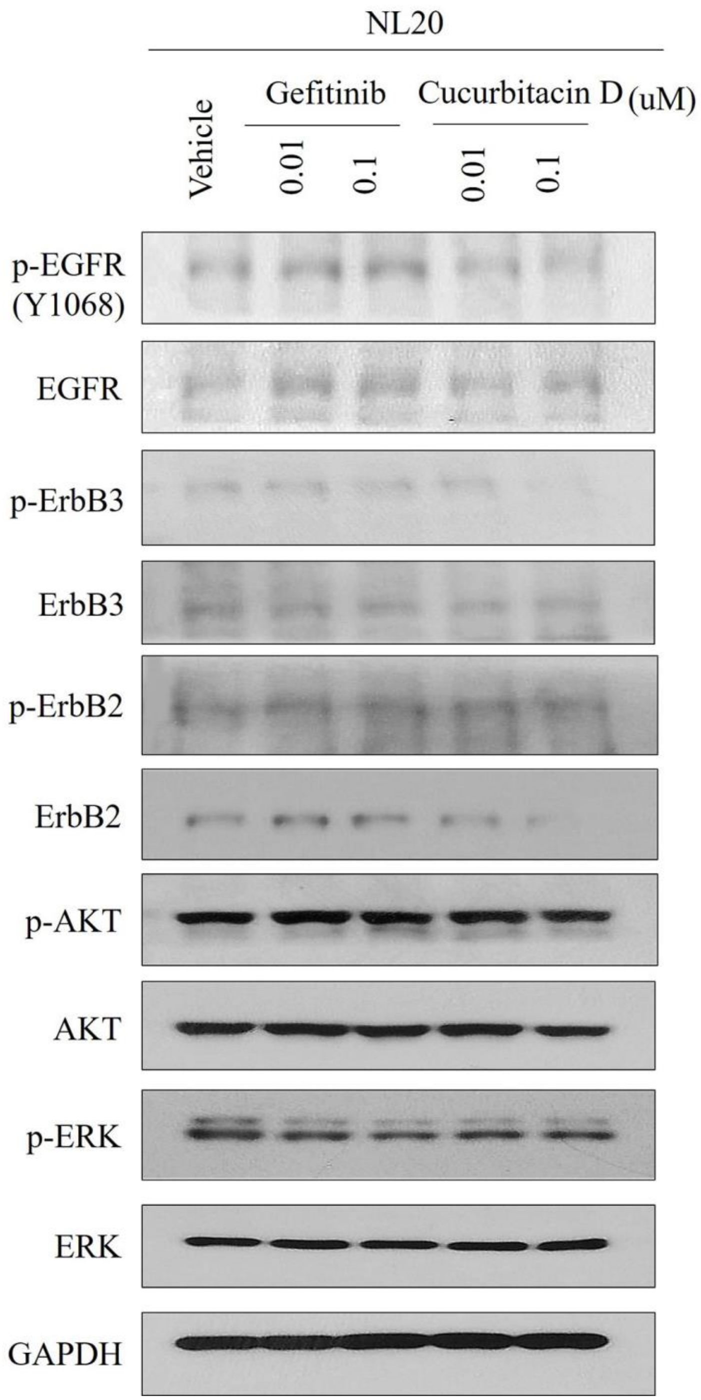

Figure S2. Measurement of EGFR signaling pathway after cucurbitacin D and gefitinib treatment. NL20 cells were treated with gefitinib and cucurbitacin D for 24 hours as indicated, Western blotting was conducted to detect the target proteins. The values shown above the blots are an analysis of the blots normalized to GAPDH.

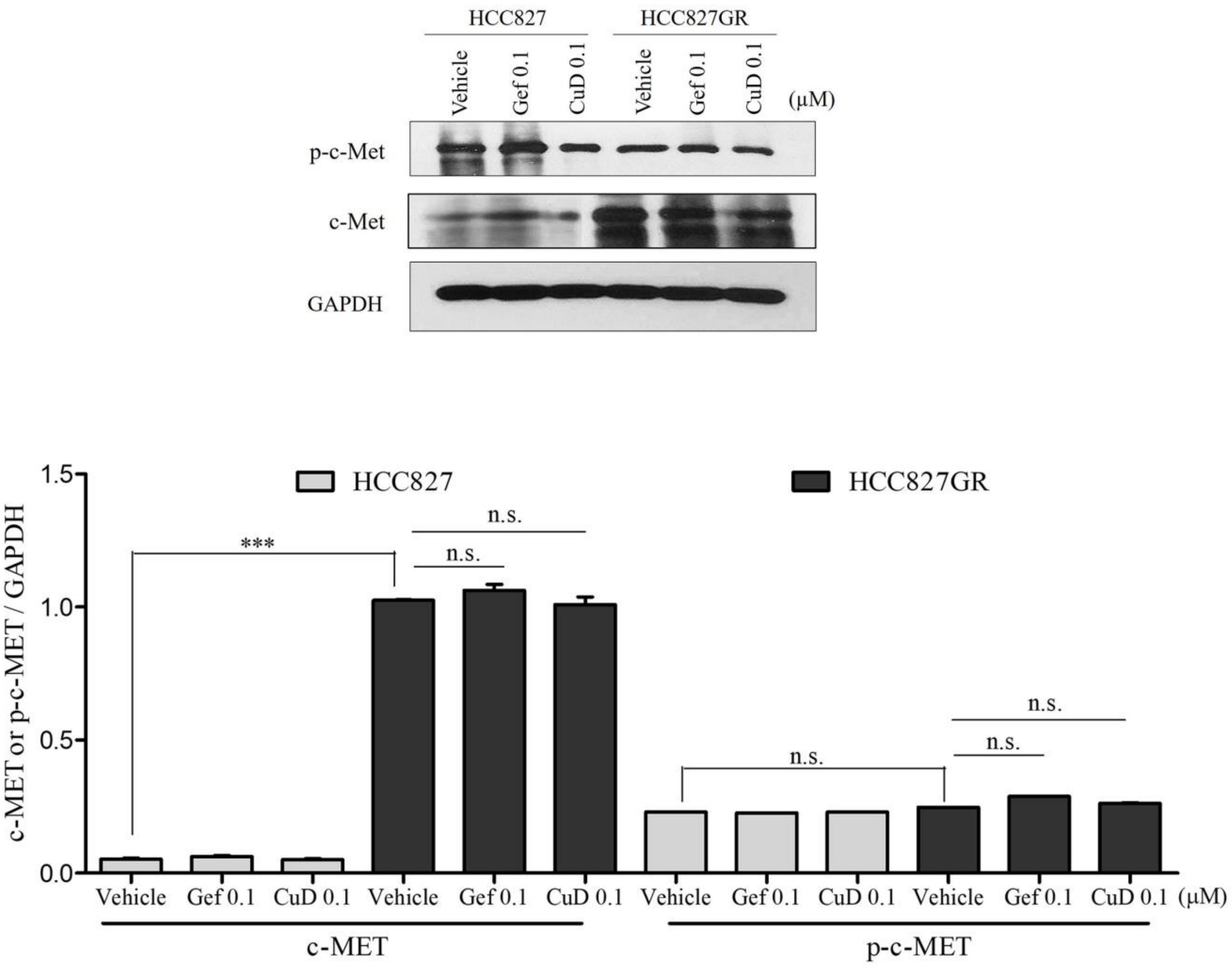

Figure S3. Measurement of MET amplification protein levels after cucurbitacin D and gefitinib treatment. HCC827 and HCC827GR cells were treated with cucurbitacin D for 24 hours as indicated, and western blotting (upper panel) was conducted to detect the target proteins (c-MET and p-c-MET). The bar graphs (lower panel) represent the quantification of the western blot data in HCC827GR cells. The values shown above the blots are an analysis of the blots normalized to GAPDH. ***$P < 0.001$. The data are presented as the mean ± S.D. CuD, cucurbitacin D; Gef, gefitinib; n.s., no significance.

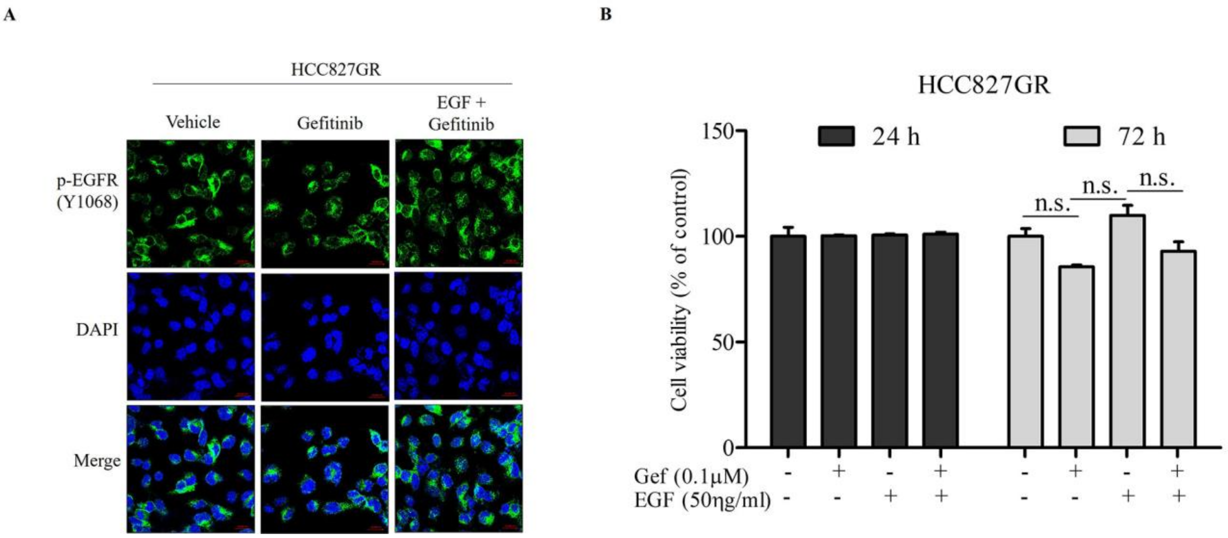

Figure S4. Measurement of intracellular EGFR accumulation and cell viability induced by gefitinib after stimulation with EGF in HCC827GR cells. (A) Immunofluorescence (IF) analysis of intracellular p-EGFR (Y1068) accumulation in the presence of EGF and gefitinib. HCC827GR cells were immunostained with a p-EGFR (Y1068) antibody (green) and counterstained with 4,6-diamidino-2-phenylindole (DAPI) (blue). Bar = 20 μm. Fluorescence microscopy images of the cell lines. (B) Cell viability in the presence of EGF and gefitinib was measured by MTT assay for 24 and 72 hours. The data are presented as the mean ± S.D. Gef, gefitinib; n.s., no significance.
